# Synthesis and Anti-Cancer Activity of the Novel Selective Glucocorticoid Receptor Agonists of the Phenylethanolamine Series

**DOI:** 10.3390/ijms25168904

**Published:** 2024-08-15

**Authors:** Ekaterina M. Zhidkova, Leyla R. Tilova, Timur I. Fetisov, Kirill I. Kirsanov, Evgeny P. Kulikov, Adel D. Enikeev, Irina V. Budunova, Gennadii A. Badun, Maria G. Chernysheva, Valerii Z. Shirinian, Marianna G. Yakubovskaya, Ekaterina A. Lesovaya

**Affiliations:** 1 Department of Chemical Carcinogenesis, Institute of Carcinogenesis, N.N. Blokhin National Medical Research Center for Oncology, Kashirskoe Shosse 24-15, Moscow 115478, Russia; zhidkova_em@mail.ru (E.M.Z.); timkatryam@yandex.ru (T.I.F.); kkirsanov85@yandex.ru (K.I.K.); mgyakubovskaya@mail.ru (M.G.Y.); 2 Faculty of Normal and Pathological Anatomy, H.M. Berbekov Kabardino-Balkarian State University, Chernyshevsky Str 173, Nalchik 3620004, Russia; uhtishkachik@mail.ru; 3 Institute of Medicine, Peoples’ Friendship University of Russia (RUDN University), Miklukho-Maklaya St. 6, Moscow 117198, Russia; 4 Faculty of Oncology, I.P. Pavlov Ryazan State Medical University, Vysokovol’tnaya Str 9, Ryazan 390026, Russia; e.kulikov@rzgmu.ru; 5 Oncogene Regulation Department, Institute of Carcinogenesis, N.N. Blokhin National Medical Research Center for Oncology, Kashirskoe Shosse 24-15, Moscow 115478, Russia; adelbufyeni@mail.ru; 6 Department of Dermatology, Feinberg School of Medicine, Northwestern University, East Chicago Avenue 303, Chicago, IL 60611, USA; i-budunova@northwestern.edu; 7 Faculty of Chemistry, M.V. Lomonosov Moscow State University, Leninskiye Gory 1, Moscow 119991, Russia; badunga@yandex.ru (G.A.B.); chernyshevamg@my.msu.ru (M.G.C.); 8 Laboratory of Heterocyclic Compounds, N.D. Zelinsky Institute of Organic Chemistry, 47, Leninsky Prospect, Moscow 119991, Russia; svbegunt@mail.ru

**Keywords:** selective glucocorticoid receptor agonists, transrepression, transactivation, CpdA-03, anti-lymphoma activity, mice

## Abstract

Glucocorticoids (GCs) are widely used for treating hematological malignancies despite their multiple adverse effects. The biological response to GCs relies on glucocorticoid receptor (GR) transrepression (TR) that mediates the anticancer effects and transactivation (TA) associated with the side effects. Selective GR agonists (SEGRAs) preferentially activating GR TR could offer greater benefits in cancer treatment. One of the well-characterized SEGRAs, 2-(4-acetoxyphenyl)-2-chloro-N-methylethylammonium-chloride (CpdA), exhibited anticancer activity; however, its translational potential is limited due to chemical instability. To overcome this limitation, we obtained CpdA derivatives, CpdA-01–CpdA-08, employing two synthetic strategies and studied their anti-tumor activity: 4-(1-hydroxy-2-(piperidin-1-yl)ethyl)phenol or CpdA-03 demonstrated superior GR affinity and stability compared to CpdA. In lymphoma Granta and leukemia CEM cell lines, CpdA-03 ligand exhibited typical SEGRA properties, inducing GR TR without triggering GR TA. CpdA-03 effects on cell viability, growth, and apoptosis were similar to the reference GR ligand, dexamethasone (Dex), and the source compound CpdA. In vivo testing of CpdA-03 activity against lymphoma on the transplantable P388 murine lymphoma model showed that CpdA-03 reduced tumor volume threefold, outperforming Dex and CpdA. In conclusion, in this work, we introduce a novel SEGRA CpdA-03 as a promising agent for lymphoma treatment with fewer side effects.

## 1. Introduction

Glucocorticoids (GCs) are essential regulators of many physiological processes, including homeostasis, embryonic and post-embryonic development, stress response, and immunomodulation [1]. Specific cytotoxic effects of GCs on a number of immune cells made them viable treatment options for blood cancer [2]. Combined GC-based therapies have remained the mainstay treatment of hematological malignancies over the last few decades. In the therapy of solid cancers, especially in the treatment of hormone-dependent prostate and breast cancer, GCs serve as an adjuvant to expand the therapeutic interval of the main cytostatic and reduce side effects from chemotherapy [3,4,5]. However, their long-term administration is limited by major debilitating side effects: skin and muscle atrophy, osteoporosis, Cushing syndrome, and steroid-induced diabetes [6,7,8,9,10,11].

Pleiotropic effects of GCs are mediated by the glucocorticoid receptor (GR), a well-known transcription factor. In the absence of a ligand, GR is located in the cytoplasm, a constituent of the multi-chaperone complex. Upon GC binding, GR modulates the transcription of the target genes through: (i) GC-responsive element (GRE)-mediated direct transactivation (TA), or (ii) transrepression (TR) of transcriptional activators such as NFκB, AP1, and STAT3 [12,13,14,15]. Therapeutic anti-cancer and anti-inflammatory activities of GCs are mediated mostly by GR TR, whereas the GC-induced metabolic complications are associated with GR TA. Selective GR agonists (SEGRAs) that shift GR function towards TR appear to be a safer treatment option for hematological malignancies. Over the past two decades, a number of SEGRAs have been discovered, synthesized, and described: mapracorat [16], CpdA [17], CpdX [15,18,19], ZK 216348 [20], AL-438 [21], Org 214007-0 [22], and AZD9567 [23]. Some of them have entered clinical trials as anti-inflammatory agents with reduced side effects [24,25]; however, none of them has yet reached clinical practice, particularly in cancer treatment.

2-(4-acetoxyphenyl)-2-chloro-N-methylethylammonium-chloride, also known as Compound A (CpdA), is a synthetic analogue of hydroxyphenyl aziridine precursor isolated from the Namibian shrub Salsola tuberculatiformis Botschantzev [26]. CpdA was shown to compete with GCs for GR binding. Since it activates GR TR without inducing GR TA, it exhibits anti-cancer and anti-inflammatory effects on different in vitro and in vivo models [11,17,27], while, in contrast to GCs, it has fewer side effects related to the function of the hypothalamic–pituitary–adrenal (HPA) axis and bone metabolism [11,27]. In water solutions, CpdA was shown to decompose to an aziridine derivative [28], referred as the 2B carcinogen according to the classification of the International Agency for Research on Cancer (IARC) [29]. Stable CpdA analogues have not been described so far.

Here, we describe the synthesis of eight CpdA chemical derivatives and evaluate their putative SEGRA cytotoxic effects. For this purpose, we employed two alternative synthetic strategies. Our findings show that one of the novel compounds, Cpda-03, exhibited the most pronounced cytotoxic effects in vitro and possessed SEGRA properties and anti-lymphoma activity both in vitro and in vivo.

## 2. Results

### 2.1. Synthesis of CpdA Chemical Derivatives

**Figure 1:**
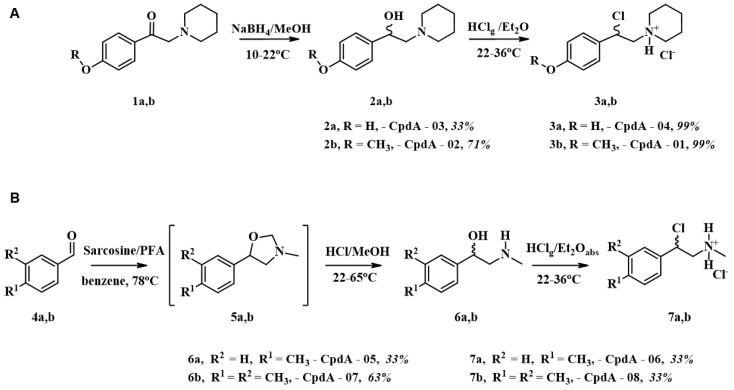
Synthesis of CpdA analogues (**A**) CpdA-01–CpdA-04; (**B**) CpdA-05–CpdA-08.

CpdA derivatives synthesized and evaluated in this work as well as the parent molecule, CpdA, are the analogues of synephrine, a natural alkaloid from citrus fruits, widely used for weight loss/weight management, controlling appetite, maintaining energy levels, enhancing sports performance, contributing to mental focus, and improving cognition [30,31]. An efficient method based on using the commercially available compounds has been proposed for the synthesis of CpdA analogues (CpdA-01–04, Figure 1). We optimized all stages of the synthesis to reduce the impurities, primarily the content of heavy metals. To synthesize CpdA-05–CpdA-08 (Figure 1), we employed the approach based on the preparation of amino alcohols by the reaction of azomethine ylide with aromatic aldehydes [32]. We optimized this protocol as well to avoid the final purification step and to improve the yields of the target compounds.

### 2.2. Screening of Cytotoxic Effects of Newly Synthesized CpdA Derivatives

We performed cytotoxicity screening for eight CpdA derivatives in three cell lines originating from hematological malignancies: acute lymphoblastic leukemia CCRF-CEM (CEM) cells, chronic myeloid leukemia K562 cells, and mantle cell lymphoma Granta-519 (Granta). All three cell lines had different sensitivity to all compounds tested, including the parent molecule CpdA and Dex used as a positive control. CEM and Granta cells characterized by elevated GR expression (Table 1) were more sensitive to the CpdA derivatives than K562 cells, which were used further only for the ligand binding assay in order to retain the maximum amount of GR for competitive binding. Among the novel CpdA derivatives, CpdA-03 exerted the most pronounced cytotoxic effects, and therefore was used for our further experiments.

### 2.3. Affinity of CpdA-03 to GR

**Figure 2:**
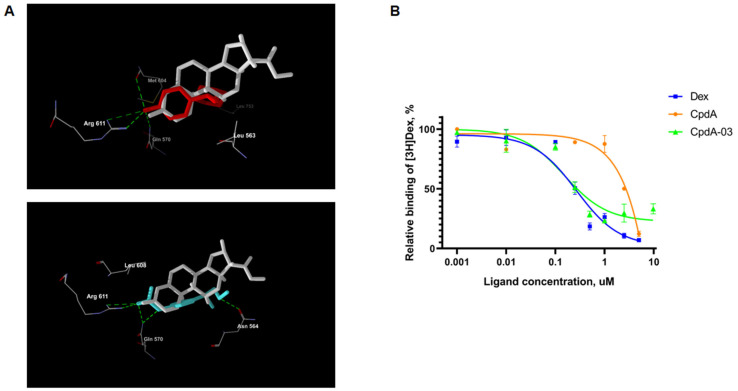
Evaluation of CpdA-03 affinity to GR. (**A**) Virtual docking of Dex (white) and CpdA-03 (red)/CpdA (light blue). (**B**) GR binding affinity was estimated using the radioligand binding assay. Dex, CpdA, and CpdA-03 in various concentrations were added to each reaction tube and incubated for 90 min. Cell lysate radioactivity was measured using a RackBeta 1215 LC (LKB Wallac, Turku, Finland) spectrometer for 4 min. Results are presented as percentage of the individual signal from [3H]Dex.

The affinity of CpdA-03 to GR proposed by virtual docking (Figure 2A) and confirmed by radioligand binding assay. For this purpose, CpdA-03 was tested in a concentration range of 0.001–10 µM. We demonstrated that binding IC_50_ for the reference GR ligands Dex and CpdA were 0.23 µM and 2.5 µM, respectively, which is in line with the literature data [33,34,35], confirming the accuracy of our results. CpdA-03 showed a relative binding affinity to GR with an IC_50_ value of 0.15 µM (Figure 2B). The obtained data indicate that CpdA-03 affinity was higher compared to the original compound and therefore is a promising agent for further detailed studies.

### 2.4. CpdA-03 Exerts Anti-Cancer Activity Both In Vitro and In Vivo

**Figure 3:**
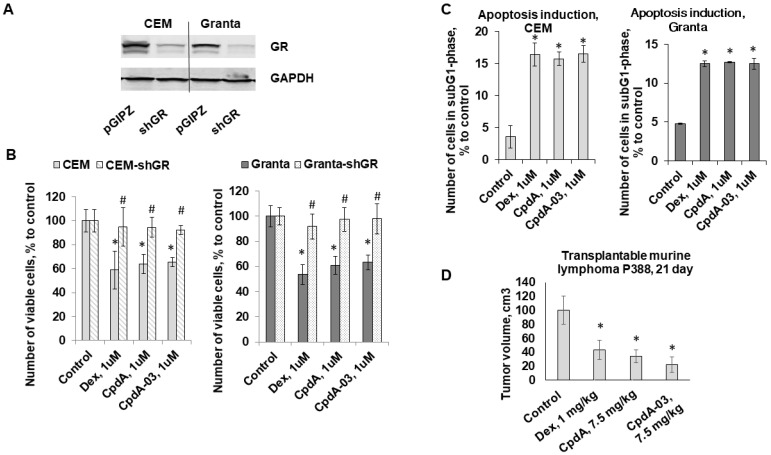
Anti-cancer activity of CpdA-03 in vitro and in vivo. GR knockdown in CEM and Granta cells was confirmed by Western blot analysis (**A**). CEM, CEM-shGR, Granta, and Granta-shGR cells were treated with a solvent (control), Dex, CpdA, or CpdA-03 (each used at 1 µM) for 24 h, and their effects on cell growth (**B**) were estimated by cell counting. Apoptosis levels (**C**) were assessed by flow cytometry using propidium iodide staining (the number of cells in the sub-G1 phase was calculated as a percentage of the entire cell populations). (**D**) In vivo anti-cancer activity was evaluated using the transplantable P388 murine lymphoma model. Animals were treated i.p. 3 times per week with Dex (1 mg/kg), CpdA (7.5 mg/kg), CpdA-03 (7.5 mg/kg), or the vehicle (5% DMSO in PBS). Tumor nodule size was measured twice a week by digital calipers. * Statistically significant difference (*p* < 0.05) compared to control, # statistically significant difference (*p* < 0.05) between control cells and GR-knockdown cells treated with the same agent.

To evaluate the anti-cancer potential of CpdA-03, we compared its effects on the proliferation of CEM and Granta cells with the original compound CpdA and the GC positive control Dex. CpdA and Dex were used at a concentration of 1 µM, following our previous work [27,36]. CpdA-03’s growth-inhibitory effect was first studied in a wide concentration range of 10 nM–10 µM in three cell lines (Table 1). In further experiments, CpdA-03 was used at a concentration of 1 µM, which is close to its IC_50_. This dose exhibited pronounced cytotoxic effects at the same concentrations as CpdA and Dex (Figure 3B). GR blockage by the shGR-expressing lentivirus (Figure 3A) resulted in a drastic loss of sensitivity to CpdA-03 as well as CpdA and Dex in both cell lines (Figure 3B). These results provide further evidence that CpdA-03 is a GR ligand and its anti-lymphoma effects are mediated by GR activation.

Next, we examined CpdA-03 effects on cell survival and found that it can induce apoptosis in both cell lines. Flow cytometry analysis showed that the number of apoptotic cells (the sub-G1-phase cell population) reached 15–17% after 48 h incubation with CpdA-03 compared to 2–4% in control (Figure 3C). These results were similar to the level of apoptosis induction caused by CpdA and Dex (Figure 3C).

For the in vivo experiment, we employed the P388 murine lymphoma model, which can be grown both subcutaneously and intraperitoneally and had been used before for estimating GC anti-cancer effects in comparison with different cytostatic drugs [37,38]. We chose subcutaneous grafting of P388 cells, which allowed for comparison of tumor growth dynamics following treatment with Dex (1 mg/kg), CpdA (7.5 mg/kg), CpdA-03 (7.5 mg/kg) or the vehicle (5% DMSO in PBS). Dex and CpdA doses were selected based on our previous studies and literature data [11,39]. I.p. injections of CpdA, Dex and CpdA-03 were started on the 11th day after P388 implantation (after the formation of the first tumor nodules). A comparative analysis of tumor volumes on the 12th day following the first injection (the 23rd day of the experiment) demonstrated a statistically significant tumor growth inhibition of 57% in the Dex group, 66% in the CpdA group, and 78% in the CpdA-03 group (Figure 3D).

### 2.5. Effects of CpdA-03 on GR Functions

**Figure 4:**
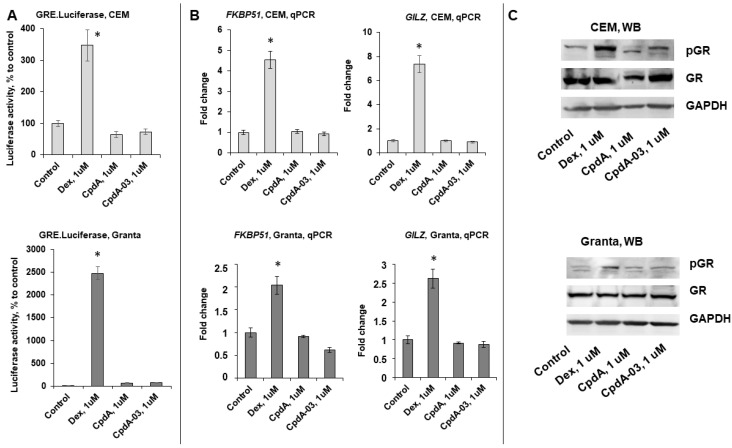
CpdA-03 does not affect GR transactivation, but induces GR transrepression in lymphoma and leukemia cells. (**A**) CEM and Granta cells stably infected with lentiviruses expressing Luciferase reporters: GRE.Luc. Cells were incubated for 24 h with the solvent (control), Dex, or CpdA. Luciferase activity was determined as described in Section 4. (**B**) Q-PCR analysis of the FKBP51 and GILZ mRNA expression in CEM and Granta cells. The Q-PCR results were normalized to the expression of the housekeeping gene RPL27 and are presented as fold change compared to control. The mean ± SD was calculated for three individual samples/condition. * Statistically significant difference (*p* < 0.05) compared to control. (**C**) The levels of phospho-GR (Ser211) and GR were determined by Western blot analysis. GAPDH was used as a loading control.

To assess the effect of CpdA-03 on GR TA function, we used CEM and Granta cells transduced with the GRE.Luc reporter. Dex and CpdA were used for comparative analysis. In contrast to Dex, neither CpdA-03 nor CpdA induced GRE Luciferase activity in either cell line (Figure 4A). Attenuation of GR TA was further confirmed by Q-PCR analysis of the known GR-target genes, including FKBP51, encoding the GR chaperone [27,40], and GILZ, which mediates GC anti-inflammatory effects [41,42] (Figure 4B), as well as by Western blot analysis of the phosphorylated GR (Ser211) content (Figure 4C).

**Figure 5:**
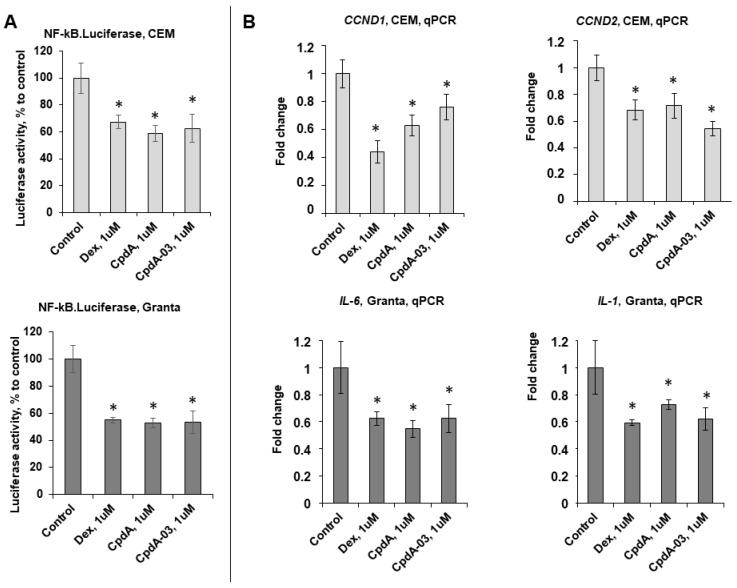
CpdA-03 induces GR transrepression in lymphoma and leukemia cells. (**A**) CEM and Granta cells stably infected with the lentiviruses bearing the NF-kB.Luciferase reporter. Cells were incubated for 24 h with the solvent (control), Dex, or CpdA. Luciferase activity was determined as described in Section 4. (**B**) CCND1 and CCND2 mRNA expression in CEM cells, IL1 and IL6 mRNA expression in Granta cells. Q-PCR results were normalized to the expression of housekeeping gene RPL27 and are presented as fold change compared to control. The mean ± SD was calculated for three individual samples/condition. * Statistically significant difference (*p* < 0.05) compared to control.

We analyzed GR TR function using the NF-kB.Luc reporter assay and found that CpdA-03 significantly reduced NF-kB activity. We also observed reduced luciferase activity in the presence of Dex and CpdA (Figure 5A), which is in line with our previous results [11,27,36]. This finding was further confirmed by Q-PCR analysis demonstrating the downregulation of several endogenous genes such as cell cycle regulator cyclins CCND1/D2 [43,44,45] in CEM cells as well as interleukins IL1 and IL6 involved in lymphoid cell survival in the Granta cell line (Figure 5B).

## 3. Discussion

The development of novel methods to alleviate GC-induced side effects is a highly relevant challenge/topic in clinical research. Two major approaches to attenuate GR TA induction and prevent metabolic and atrophic complications involve using a combination of GCs with compounds protecting tissues against adverse steroid effects [40,45,46] and designing SEGRAs that shift GR activity towards TR [15,16,17,18,19,21,22,23,47].

Previous studies by our research group were focused on the anti-cancer effects of Compound A (CpdA), a unique non-steroidal SEGRA that is a stable synthetic analogue of a compound found in the Namibian shrub Salsola tuberculatiformis Botschantzev [26]. However, CpdA is highly labile and is decomposed in an aqueous medium into the mutagen aziridine and then polyphenol synephrine [28]. Although CpdA is stabilized by binding to plasma proteins and remains biologically active in vivo [28], its degradation can occur during storage and preparation of medication, thereby potentially adversely affecting its activity. The advantage of the approach developed for the synthesis of CpdA analogues (CpdA-01–CpdA-08) lies in using the commercially available compounds without metal-containing catalysts and toxic solvents.

Here, we modified the CpdA molecule in order to increase its stability while retaining the relative negative electron charges that facilitate the formation of H-bonds with several amino acids lining the binding cavities of GR (Asn564 and Arg611) [34]. The virtual docking results confirmed that CpdA and its derivatives shared the binding cavity (Figure 2A, Supplementary Figure S1). The MolDock score for all eight CpdA derivatives was comparable to the MolDock score of the prototype CpdA. Therefore, the main criterion for the selection of the leader compound was the GR-dependent cytotoxic effect, which was demonstrated for CpdA-03 (Figure 3).

The most promising CpdA derivative, CpdA-03, was an analogue of alkaloid synephrine, incapable of forming an aziridine derivative due to the absent proton at the nitrogen atom. We performed a stability assay of CpdA-03 for 1 month under normal conditions and demonstrated the stability of this molecule (Supplementary Figure S2). CpdA-04, the CpdA derivative with lower affinity to GR, was a more hydrophilic synephrine analogue. CpdA-01, CpdA-02, and CpdA-05–CpdA-08 were characterized by a large number of substituting groups, which impeded their binding with Asn564 and Arg611 at the GR active center.

Both in vitro and in vivo, CpdA-03 exhibited anti-cancer activity comparable to Dex and CpdA (Figure 3 [11,27]). The anti-cancer effect of CpdA-03 as a GR ligand could be associated with GR TR activation, which was shown in the NF-kB.Luciferase assay (Figure 5A). Moreover, CpdA-03 is less likely to induce therapeutic resistance, as it did not activate FKBP51, the direct GR target gene [48], which is involved in the feedback control of GR signaling via GR retention in the cytoplasm and associated with GC resistance in patients with brain and prostate cancer, melanomas, and lymphomas [49,50].

In conclusion, we report the novel SEGRA 4-(1-hydroxy-2-(piperidin-1-yl)ethyl)phenol (CpdA-03), possessing the therapeutic anti-cancer potential of GCs, and more importantly, of the parent compound CpdA, while lacking GR TA activity that causes side effects and therapy resistance. Given its stability, these properties make CpdA-03 an attractive candidate for further research into its applications for treatment of hematological malignancies.

## 4. Materials and Methods

### 4.1. Chemistry

#### 4.1.1. General Information

The structures of compounds were determined by ^1^D NMR (^1^H, ^13^C) spectroscopy (see Supplementary Figures S3–S10 for the spectra). NMR spectra were registered on Bruker 300 spectrometers (Billerica, MA, USA) at 293; chemical shifts were measured in ppm relative to the solvent (^1^H: DMSO-d_6_, d 2.50 ppm; ^13^C: DMSO-d_6_, d 39.50 ppm). The splitting patterns are designated as s, singlet; d, doublet; t, triplet; q, quartet; m, multiplet; dd, double doublet; ddd, double double doublet; dt, doublet triplet. The coupling constants (J) are in hertz. High-resolution mass spectra (HRMS) were obtained using electrospray ionization (ESI) in positive ion mode (interface capillary voltage 4500 V); the mass range was from *m*/*z* 50 to 3000 Da; external/internal calibration was performed using an electrospray calibration solution (see HRMS for CpdA-01 and CpdA-08, Supplementary Figures S11 and S12). A syringe injection was used for solutions in CH_3_CN (flow rate 3 mL/min). Melting points were measured using a Boetius capillary melting point apparatus and are uncorrected. Analytical thin-layer chromatography (TLC) was carried out on silica gel plates (silica gel 60 F254 aluminum-supported plates); a UV lamp (254/365 nm) was used for the visualization. All chemicals and anhydrous solvents were purchased from commercial sources and used without further purification. Silica column chromatography was performed on silica gel 60 (70–230 mesh) and petroleum ether–ethyl acetate–Et_3_N (1:1:0.5) as eluent; TLC analysis was conducted on silica gel 60 F254 plates.

#### 4.1.2. Synthesis and Characterization of CpdA-01–CpdA-04

Amino alcohol 2 was obtained by reduction of ketone 1 with sodium borohydride (Figure 1). The target compound 3 was obtained by the reflux of the previously synthesized amino alcohol in a stream of dry hydrogen chloride in absolute diethyl ether. The characteristics of CpdA piperidine analogues are summarized in Supplementary Table S1.

Stage 1. Sodium borohydride (30 mmol) was added portion-wise to a solution of aminoketone 1 (3.0 mmol), cooled to 10 °C in abs. methanol (30 mL), and the solution was stirred at room temperature for 5 h. The reaction mixture was introduced into water (150 mL) and extracted with ethyl acetate (3 × 30 mL); the extract was washed with water (50 mL) and evaporated. The residue was purified by chromatography (petroleum ether–EtOAc–Et_3_N 1:1:0.05), and the diastereomers were not separated. The residue was recrystallized from a methylene chloride–heptane mixture.

Stage 2. Dry hydrogen chloride was passed through a solution of amino alcohol 2 (3.0 mmol) in abs. diethyl ether (60 mL) with stirring, after which the reaction mixture was refluxed for 20 h. After cooling the reaction mixture, the white precipitate obtained was filtered off and thoroughly washed with absolute diethyl ether. The residue was dried under vacuum at room temperature.

#### 4.1.3. Synthesis and Characterization of CpdA-05–CpdA-08

These analogues of CpdA were prepared following a previously reported method [51] from the corresponding aromatic aldehydes (Figure 1). The characteristics of CpdA-05–CpdA-08 are summarized in Supplementary Table S1.

A mixture of the corresponding aromatic aldehyde (1.0 mmol), finely ground sarcosine (0.13 g, 1.5 mmol), and paraformaldehyde (0.09 g, 3.0 mmol) was refluxed in dry benzene (3.3 mL), with magnetic stirring and removal of the formed water by a Dean–Stark trap for 6–8 h. The resulting solution was evaporated in vacuo, providing the oily 5-aryl-3-methyloxazolidines. Oxazolidine 5 was dissolved in MeOH (1 mL) and treated with concentrated HCl (0.10 mL, 1.2 mmol). The resulting mixture was refluxed under a fume hood with partial evaporation of the solvent for 1.5 h (for removal of dimethoxymethane). MeOH was evaporated in vacuo, and H_2_O (0.5 mL) was added. The mixture was extracted with Et_2_O (2 × 1 mL) followed by basification with an excess of a cold concentrated solution of NaOH. Extraction with CH_2_Cl_2_ (2 × 2 mL), drying over Na_2_SO_4_, and evaporation produced the crude 1-aryl-2-(methylamino)ethanol, which was recrystallized from a CH_2_Cl_2_–heptane mixture. Ammonium salts 7a,b were obtained as described above.

#### 4.1.4. CpdA-03 Stability

CpdA-03 (30 mg) was suspended in 800 μL of D_2_O with 6.3 mg of sodium metal pre-dissolved in it. Then, ^1^H NMR spectra were recorded: the first immediately after sample preparation, the second after 1 month’s storage of the solution at room temperature. Conclusions about the test compound stability were made by comparing the spectra (Supplementary Figure S2).

### 4.2. Biology

#### 4.2.1. Cell Cultures and Treatments

The acute lymphoblastic leukemia cell line CCRF-CEM, chronic myeloid leukemia cell line K562, and mantle cell lymphoma cell line Granta-519 (hereafter CEM, K562, and Granta) were obtained from the ATCC and cultured as described [27]. Cells were treated with dexamethasone (Dex), CpdA, CpdA-01–CpdA-08, or vehicle (0.01% DMSO) for 24 h.

#### 4.2.2. MTT Cytotoxicity Assay

Cells were seeded in 96-well plates (10^4^ cells/well) and incubated overnight under 5% CO_2_ at 37 °C. Next, cells were treated with Dex, CpdA, CpdA-01–CpdA-08 (0.1–100 μM) or vehicle for 24 h. Then, 20 μL of the MTT solution was added to each well and mixed. After 4 h, the supernatants were removed and 100 μL DMSO was added to each well to dissolve the precipitate. Cell viability was estimated by measuring absorbance at 570 nm using a MultiScan MCC 340 spectrophotometer (Thermo Fisher, Waltham, MA, USA). The IC_50_ values for each cell line were determined using GraphPad Prism 7.0. Results are presented as means ± standard deviation (SD).

#### 4.2.3. Western Blot Analysis

Western blot analysis was performed as described previously [27,52]. Proteins were resolved by SDS-PAGE. Membranes were blocked with 5% nonfat milk solution and then incubated with primary antibodies against GR (H-300) and phospho-GR (pGR) (Santa Cruz Biotechnology, Dallas, TX, USA) overnight at 4 °C. To verify equal protein loading and adequate transfer, membranes were probed with GAPDH antibodies (Cell Signaling, Danvers, MA, USA). Goat anti-rabbit IgGs (Jackson Immuno Research, West Grove, PA, USA) were used as secondary antibodies. Signals were detected using the ECL reagent and an ImageQuant LAS4000 system (GE HealthCare, Chicago, IL, USA). ImageJ software version 1.54f (NIH) was used for densitometry.

#### 4.2.4. Cell Viability

Cell viability was measured by direct cell counting. Cells were plated in 24-well plates (10^4^ cells/well) and cultured in complete medium in the presence of CpdA, CpdA-03, Dex, or vehicle for 24 h.

#### 4.2.5. Cell Cycle Analysis

Cell cycle analysis was performed by propidium iodide (PI) staining, as described in [27]. Cells were fixed in 70% ethanol for 2h at 4 °C, resuspended in PBS containing 50 mg/mL PI, 0.1% sodium citrate and 0.3% NP-40, and incubated for 30 min at room temperature. Cells were analyzed by a FACScan flow cytometer (Becton Dickinson, Franklin Lakes, NJ, USA) to discriminate between live and apoptotic cells.

#### 4.2.6. RNA Extraction and Q-PCR

Total RNA isolation, reverse transcription, and Q-PCR were performed as described in [45]. Primers were designed with NCBI Primer-BLAST (Supplementary Table S1). Results were normalized to the expression of the housekeeping RPL27 gene [45,46]

#### 4.2.7. Lentiviral Technology

Lentiviral stocks were obtained using the lentiviral expression vectors obtained from Northwestern University SDRC DNA/RNA Delivery Core, as described previously [27,52]: pGIPZ encoding short hairpin RNA (shRNA) against GR or the empty vector used as a control (Open Biosystems, Huntsville, AL, USA); NF-kB.Luc and GRE.Luc encoding firefly luciferase reporters or firefly luciferase under a minimal CMV promoter used as control (System Biosciences, Palo Alto, CA, USA). Lymphoma cell lines stably infected with lentiviruses were selected with 0.5 ug/mL puromycin.

#### 4.2.8. Luciferase Assay

Cells stably expressing firefly luciferase under NF-kB or GRE promoters were seeded in a 24-well plate (10^4^ cells/well) and treated with CpdA, CpdA-03, Dex, or the vehicle for 24 h. Luciferase activity was measured as described in [45,46] using the commercial Luciferase Assay (Promega, Madison, WI, USA) and Luminometer TD 20/20 (Turner Design Instruments, San Jose, CA, USA). Cells stably expressing firefly luciferase under minimal CMV promoter were used as a control to adjust for non-specific toxicity. The results were normalized by total protein amount in each sample.

#### 4.2.9. GR Binding Affinity Assay

The K562 cells (2.5 million cells/mL) were cultured in serum-free culture medium in the presence of 0.5 µM [3H]Dex and unlabeled Dex, CpdA, or CpdA-03 in various concentrations. After 90 min of incubation, cells were washed twice by centrifugation in cold serum-free medium (1500 rpm, 5 min). Next, cells were lysed with RIPA buffer (50 mM Tris (pH 8.0) 150 mM NaCl, 1 mM ethylenediaminetetraacetic acid, 0.1% sodium dodecyl sulfate, 0.5% sodium deoxycholate, 1% triton-X100, 10% glycerin, protease inhibitor) for 20 min. Lysates were transferred to scintillation liquid, and radioactivity was measured by a RackBeta 1215 LC spectrometer for 4 min. The relative binding percentages of Dex, CpdA, and CpdA-03 were estimated. IC_50_ was determined using GraphPad Prism 7.0. Measured values, presented in dpm (decay per minute) of different Dex concentrations, were calculated using non-linear regression (curve fit) with equation ([Inhibitor] vs. response with three parameters: “top”, “bottom”, and IC50). “Top” indicated the highest value in dpm and was equal to 100%, while “bottom” was considered the lowest value (0%). Dpm for CpdA and CpdA-03 compounds was calculated in the same way using the “bottom” of Dex in the individual biological repeat. These calculations produced plotting curves, precisely fitting the pattern of competitive inhibition by a ligand of binding [3H]Dex to GR. The IC_50_ values for each ligand were determined based on the plotting of these curves.

#### 4.2.10. Anti-Cancer Study In Vivo

The protocols for the experiments involving mice were approved by the Animal Care and Use Committee of the N.N. Blokhin National Research Center of Oncology and adhered to the guidelines for the welfare and use of animals in cancer research adopted by the United Kingdom Coordinating Committee on Cancer Prevention Research [53]. P388 cells were injected i.p. in 5-week-old female DBA/2 mice (Stolbovaya Farm, Moscow region, Russia, http://www.scbmt.ru). After 15 days, cells were collected from the peritoneum, washed, and resuspended in PBS. For experiments, 0.1 mL of a suspension containing 2 million cells obtained from the ascites was inoculated s.c. in BDF1 mice (Stolbovaya Farm, Moscow region, Russia, http://www.scbmt.ru). Mice were randomly divided into four groups, with 10 animals per group, and treated i.p. 3 times/week with Dex (1 mg/kg), CpdA (7.5 mg/kg), CpdA-03 (7.5 mg/kg), or the vehicle (1% DMSO in PBS). Tumor size was measured twice a week by digital calipers.

#### 4.2.11. Virtual Docking

Molegro Virtual Docker 6.0 software was used to perform virtual docking. The structure of the GR (PDB ID: 1P93) was chosen as a target. The target structure was prepared automatically using standard procedures of the Molegro Virtual Docker package. Ligand structures were constructed and optimized by molecular dynamic methods in the MMFF94 force field using the Avogadro 1.2.0. MolDock Score was chosen as the scoring function; dexamethasone (CID 5743) served as the reference ligand. Molecular docking was carried out in 40 iterations. MolDock SE was chosen as the docking algorithm following energy minimization and optimization of hydrogen bonds.

#### 4.2.12. Statistical Analysis

Mean and standard deviation values were calculated using Microsoft Excel 2016 and GraphPad Prism version 7.0. Treatment effects in each experiment were compared by one-way ANOVA or *t*-test. Differences between groups were considered significant at *p* < 0.05. All experiments were repeated three times. In animal experiments, we used 10 animals per experimental group.

## Figures and Tables

**Table 1:** Cytotoxicity of novel CpdA derivatives in MTT-test.

Compound	IC_50_, µM, CEM	IC_50_, µM, Granta	IC_50_, µM, K562
Dex	1.4 ± 0.48	3.6 ± 0.69	8.9 ± 0.91
CpdA	1.4 ± 0.27	5.6 ± 0.27	14.6 ± 1.27
CpdA-01	15.3 ± 2.44	19.6 ± 2.85	25.0 ± 3.49
CpdA-02	18.3 ± 2.71	26.5 ± 1.84	38.7 ± 5.92
CpdA-03	3.3 ± 0.22	5.8 ± 0.63	18.8 ± 1.62
CpdA-04	24.3 ± 3.34	28.7 ± 3.50	34.4 ± 3.63
CpdA-05	9.7 ± 0.51	11.7 ± 2.01	25.8 ± 3.17
CpdA-06	9.1 ± 1.78	19.3 ± 1.79	33.9 ± 5.17
CpdA-07	18.6 ± 1.62	20.6 ± 2.02	39.9 ± 3.68
CpdA-08	27.9 ± 3.94	19.9 ± 2.56	47.4 ± 5.47

## Data Availability

The original contributions presented in the study are included in the article and Supplementary Materials, further inquiries can be directed to the corresponding author.

## Supplementary Materials

The following supporting information can be downloaded at https://www.mdpi.com/article/10.3390/ijms25168904/s1.
