# Supplementary material for: Synthesis and Anti-Cancer Activity of the Novel Selective Glucocorticoid Receptor Agonists of the Phenylethanolamine Series
Source: Int J Mol Sci. 2024 Aug 15;25(16):8904. doi: 10.3390/ijms25168904 (PMC11354514; doi:10.3390/ijms25168904)
Supplement: Supplementary file 1 [file ijms-25-08904-s001.zip › Zhidkova et al Supplementary Figure 1 Revised.pdf]

CpdA

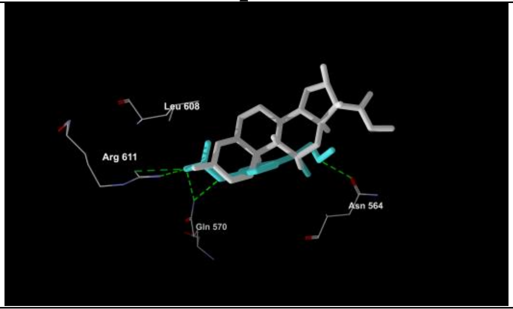

CpdA-01

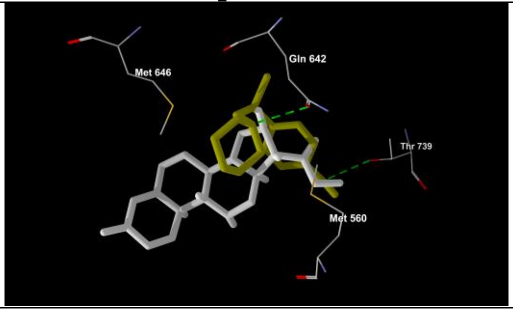

CpdA-02

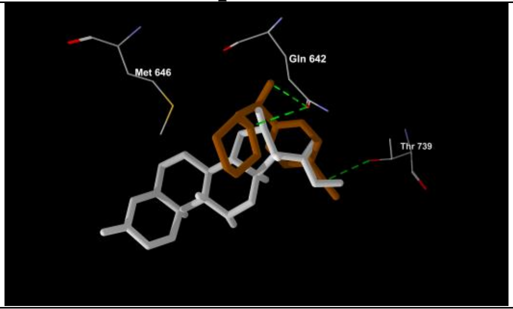

CpdA-03

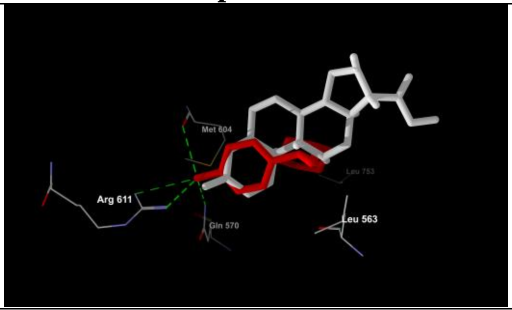

CpdA-04

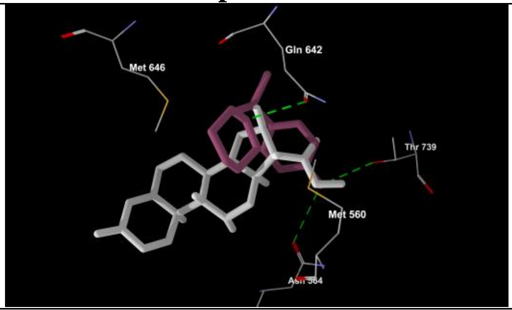

CpdA-05

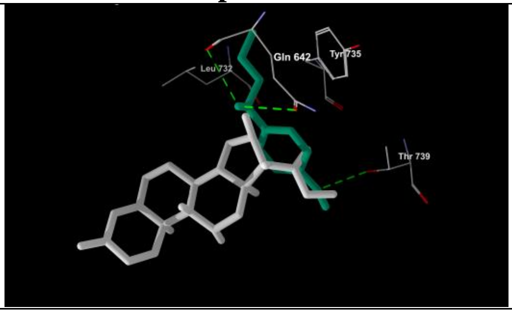

CpdA-06

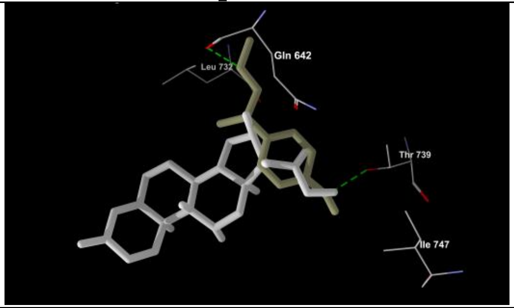

CpdA-07

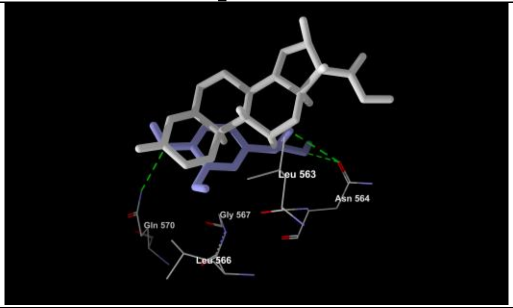

CpdA-08

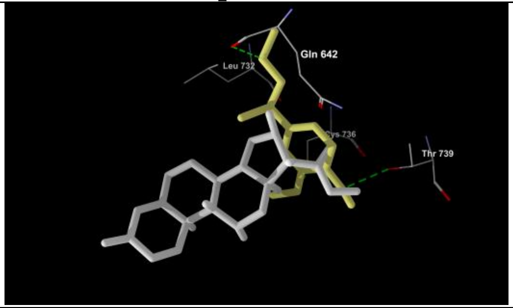

Supplementary Figure 1. Virtual docking of Dex and CpdA/CpdA-01-08. Molegro Virtual Docker 6.0 software was used to perform virtual docking. The structure of the GR (PDB ID: 1P93) was chosen as a target. The target structure was prepared automatically using standard procedures of the Molegro Virtual Docker package. Ligand structures were constructed and optimized by molecular dynamics methods in the MMFF94 force field using the Avogadro 1.2.0. MolDock Score was chosen as the scoring function; dexamethasone (CID 5743) served as the reference ligand. Molecular docking was carried out in 40 iterations. MolDock SE was chosen as the docking algorithm with following energy minimization and optimization of hydrogen bonds.
