# Supplementary material for: Synthesis and Anti-Cancer Activity of the Novel Selective Glucocorticoid Receptor Agonists of the Phenylethanolamine Series
Source: Int J Mol Sci. 2024 Aug 15;25(16):8904. doi: 10.3390/ijms25168904 (PMC11354514; doi:10.3390/ijms25168904)

Display Report

Analysis Info

Analysis Name	D:\Data\Chizhov\Zavarzin\Eskov\esk-54_&clblow.d
Method	tune_low.m
Sample Name	/VZOL esk-54
Comment	CH3CN 100 %, dil. 200, calibrant added
Acquisition Date	27.10.2014 14:08:52
Operator	BDAL@DE
Instrument / Ser#	micrOTOF 10248

Acquisition Parameter

Source Type	ESI	Ion Polarity	Positive	Set Nebulizer	0.4 Bar
Focus	Not active			Set Dry Heater	180 °C
Scan Begin	50 m/z	Set Capillary	4500 V	Set Dry Gas	4.0 l/min
Scan End	3000 m/z	Set End Plate Offset	-500 V	Set Divert Valve	Waste

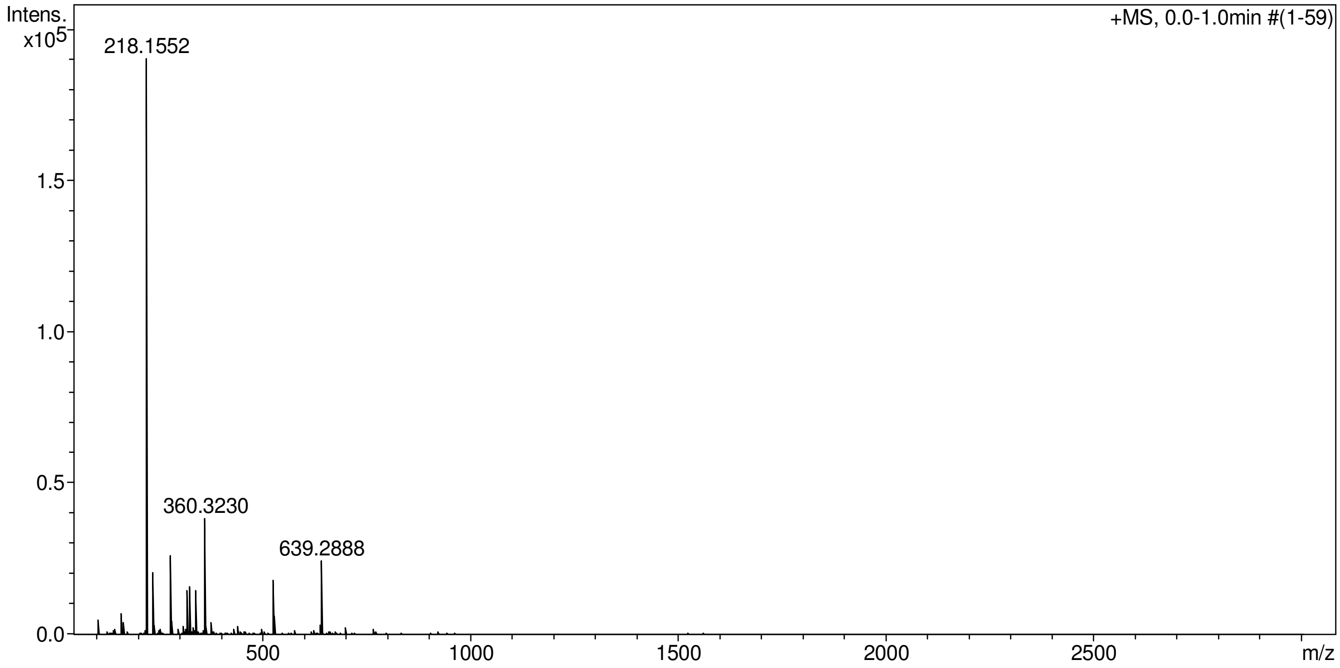

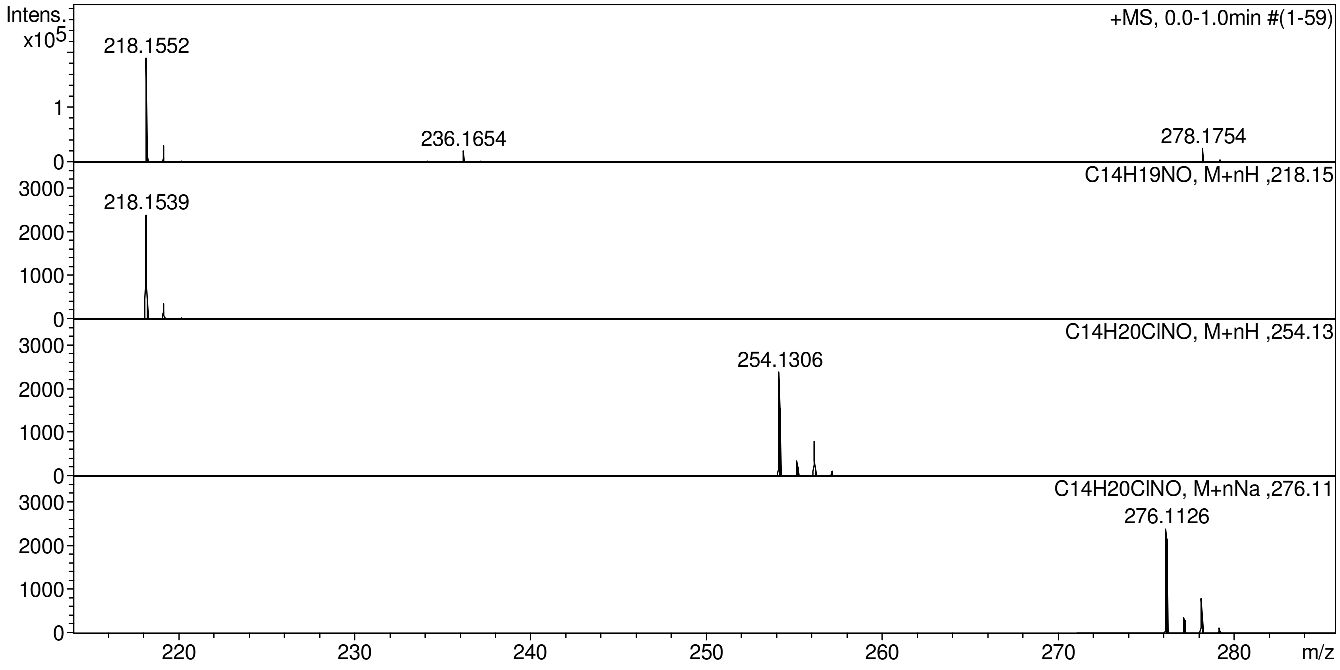

Display Report

Analysis Info

Analysis Name	D:\Data\Chizhov\Zavarzin\Eskov\esk-54_&clblow.d	Acquisition Date	27.10.2014 14:08:52
Method	tune_low.m	Operator	BDAL@DE
Sample Name	/VZOL esk-54	Instrument / Ser#	micrOTOF 10248
Comment	CH3CN 100 %, dil. 200, calibrant added		

Acquisition Parameter

Source Type	ESI	Ion Polarity	Positive	Set Nebulizer	0.4 Bar
Focus	Not active			Set Dry Heater	180 °C
Scan Begin	50 m/z	Set Capillary	4500 V	Set Dry Gas	4.0 l/min
Scan End	3000 m/z	Set End Plate Offset	-500 V	Set Divert Valve	Waste

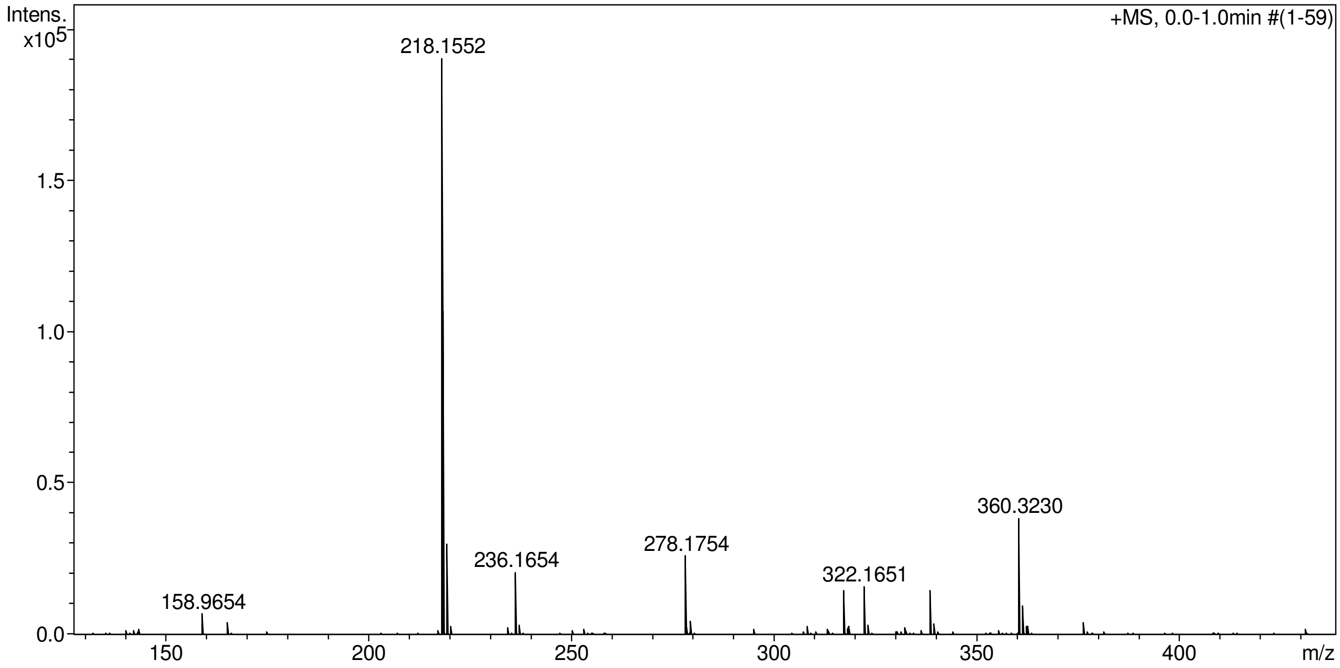

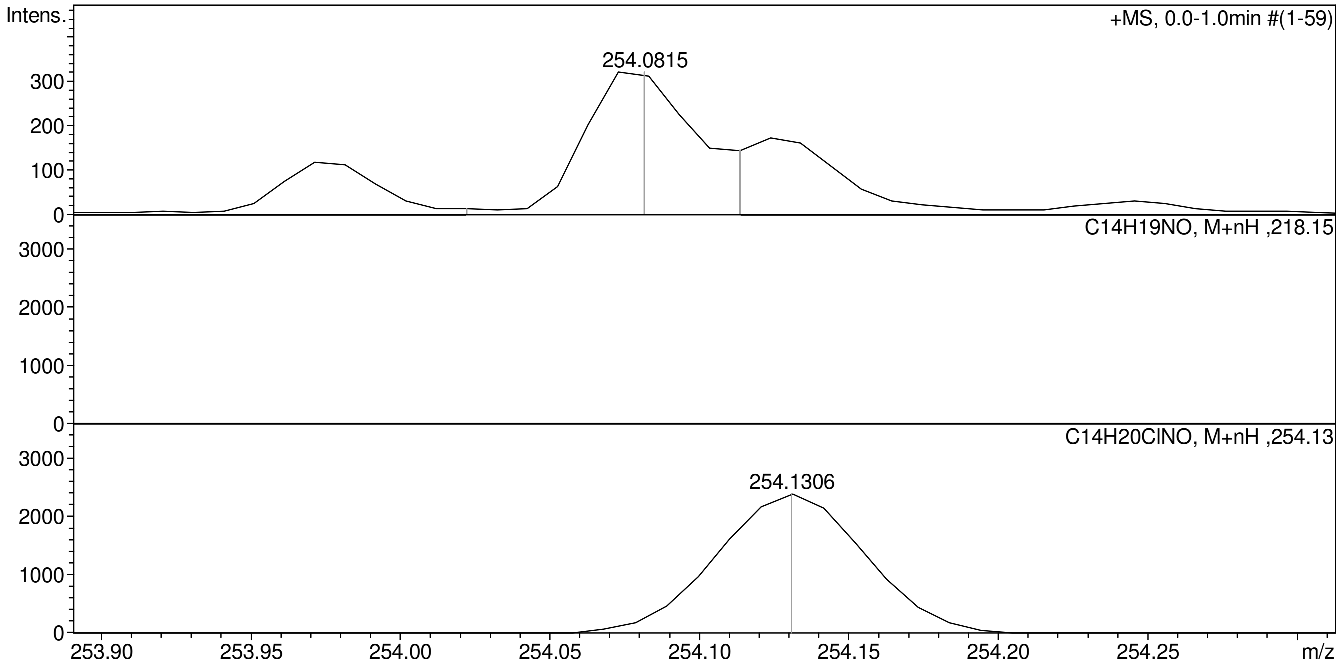

Display Report

Analysis Info

Analysis Name	D:\Data\Chizhov\Zavarzin\Eskov\esk-54_low.d	Acquisition Date	27.10.2014 14:30:32
Method	tune_low.m	Operator	BDAL@DE
Sample Name	/VZOL esk-54	Instrument / Ser#	micrOTOF 10248
Comment	CH3CN 100 %, dil. 200, no calibrant added		

Acquisition Parameter

Source Type	ESI	Ion Polarity	Positive	Set Nebulizer	0.4 Bar
Focus	Not active			Set Dry Heater	180 °C
Scan Begin	50 m/z	Set Capillary	4500 V	Set Dry Gas	4.0 l/min
Scan End	3000 m/z	Set End Plate Offset	-500 V	Set Divert Valve	Waste

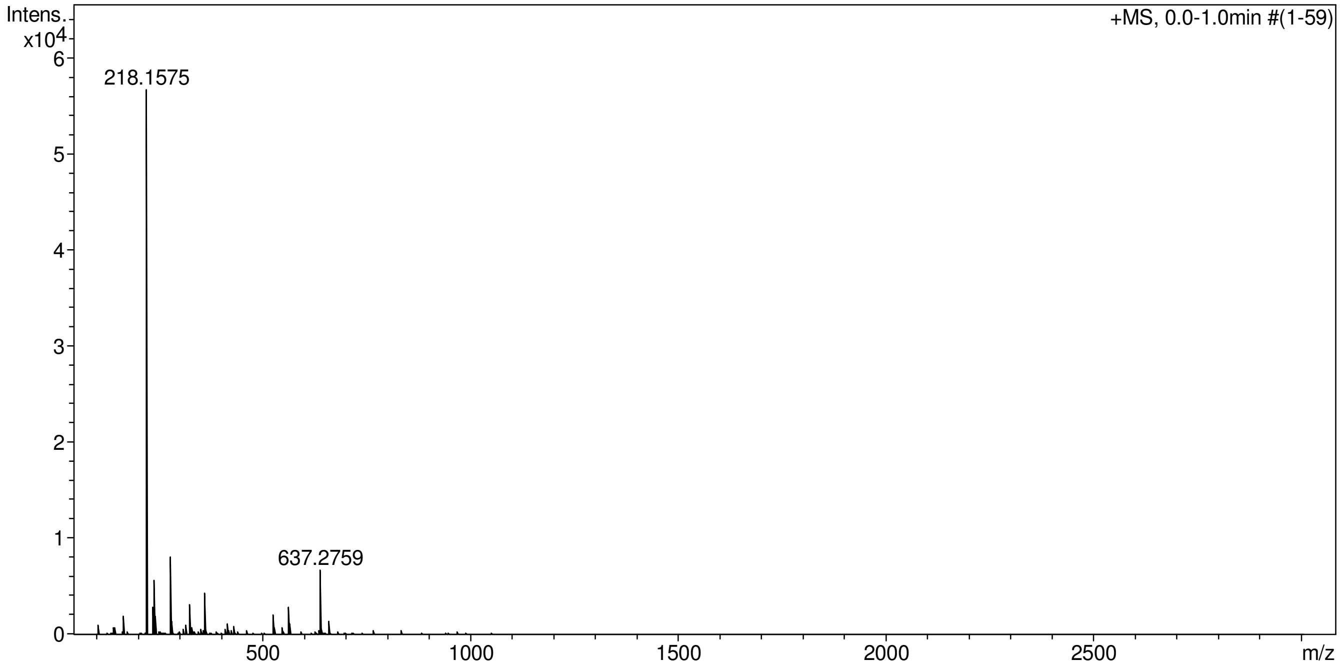

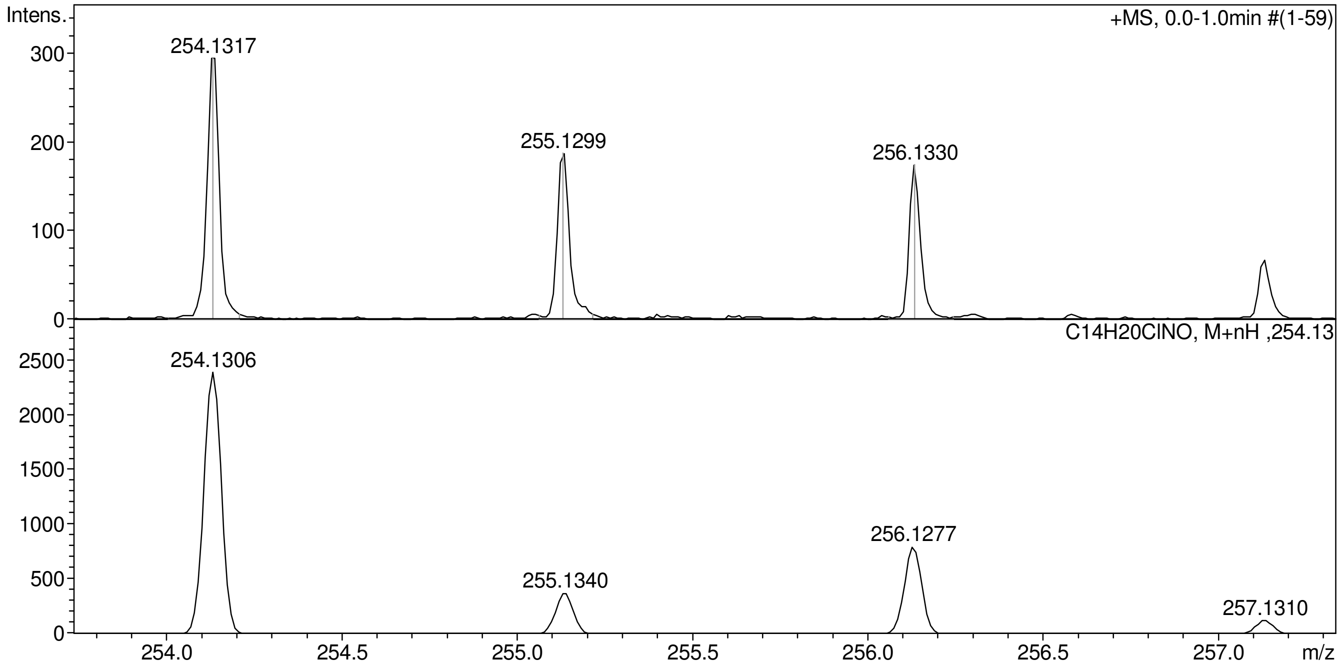

Supplement: Supplementary file 1 [file ijms-25-08904-s001.zip › Zhidkova et al Supplementary Figure 11 Revised.pdf]
