# Supplementary material for: Synthesis and Anti-Cancer Activity of the Novel Selective Glucocorticoid Receptor Agonists of the Phenylethanolamine Series
Source: Int J Mol Sci. 2024 Aug 15;25(16):8904. doi: 10.3390/ijms25168904 (PMC11354514; doi:10.3390/ijms25168904)

Display Report

Analysis Info

Analysis Name	D:\Data\Kolotyrkina\2015\Es'kov\0122004.d	Acquisition Date	22.01.2015 16:25:42
Method	tune_low.m	Operator	BDAL@DE
Sample Name	/ESKV ESK-71	Instrument / Ser#	micrOTOF 10248
Comment	C11H17NO3 mw 211 calibrant added		

Acquisition Parameter

Source Type	ESI	Ion Polarity	Positive	Set Nebulizer	0.4 Bar
Focus	Not active			Set Dry Heater	180 °C
Scan Begin	50 m/z	Set Capillary	4500 V	Set Dry Gas	4.0 l/min
Scan End	3000 m/z	Set End Plate Offset	-500 V	Set Divert Valve	Waste

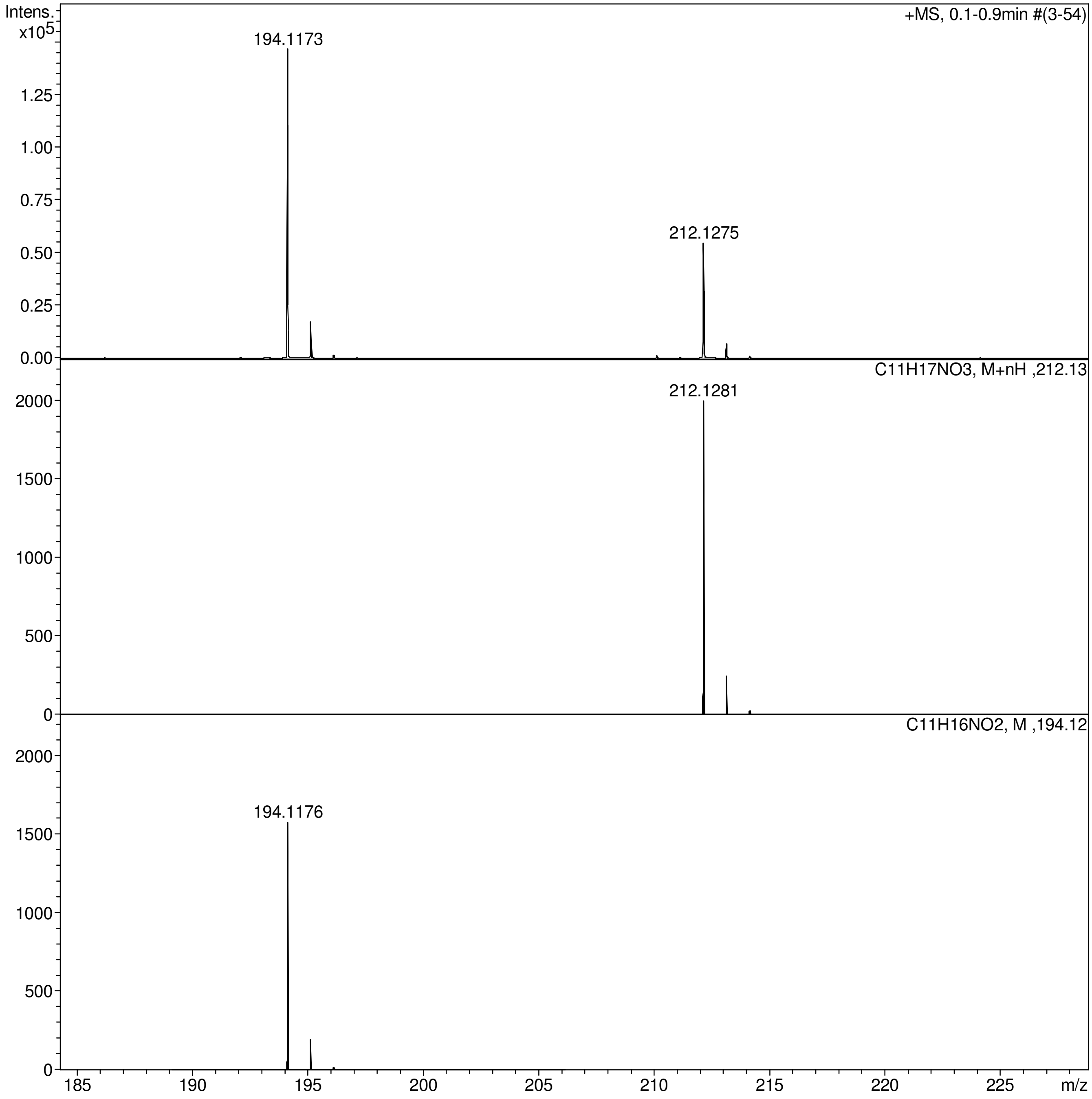

Supplement: Supplementary file 1 [file ijms-25-08904-s001.zip › Zhidkova et al Supplementary Figure 12 Revised.pdf]
