# Supplementary material for: Synthesis and Anti-Cancer Activity of the Novel Selective Glucocorticoid Receptor Agonists of the Phenylethanolamine Series
Source: Int J Mol Sci. 2024 Aug 15;25(16):8904. doi: 10.3390/ijms25168904 (PMC11354514; doi:10.3390/ijms25168904)

FW	221.2955	***Formula***	$C_{13}H_{19}NO_2$

Acquisition Time (sec)	3.4210	***Comment***	Imported from UXNMR.		
Date	09 Apr 2019 17:16:00			***File Name***	D:\Институт\Спектры_5201001r
Frequency (MHz)	300.13	***Nucleus***	1H	***Number of Transients***	8
Original Points Count	16384	***Points Count***	131072	***Pulse Sequence***	zg30
Solvent	DMSO-D6	***Sweep Width (Hz)***	4789.27	***Temperature (degree C)***	26.160

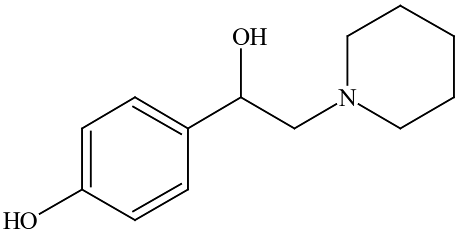

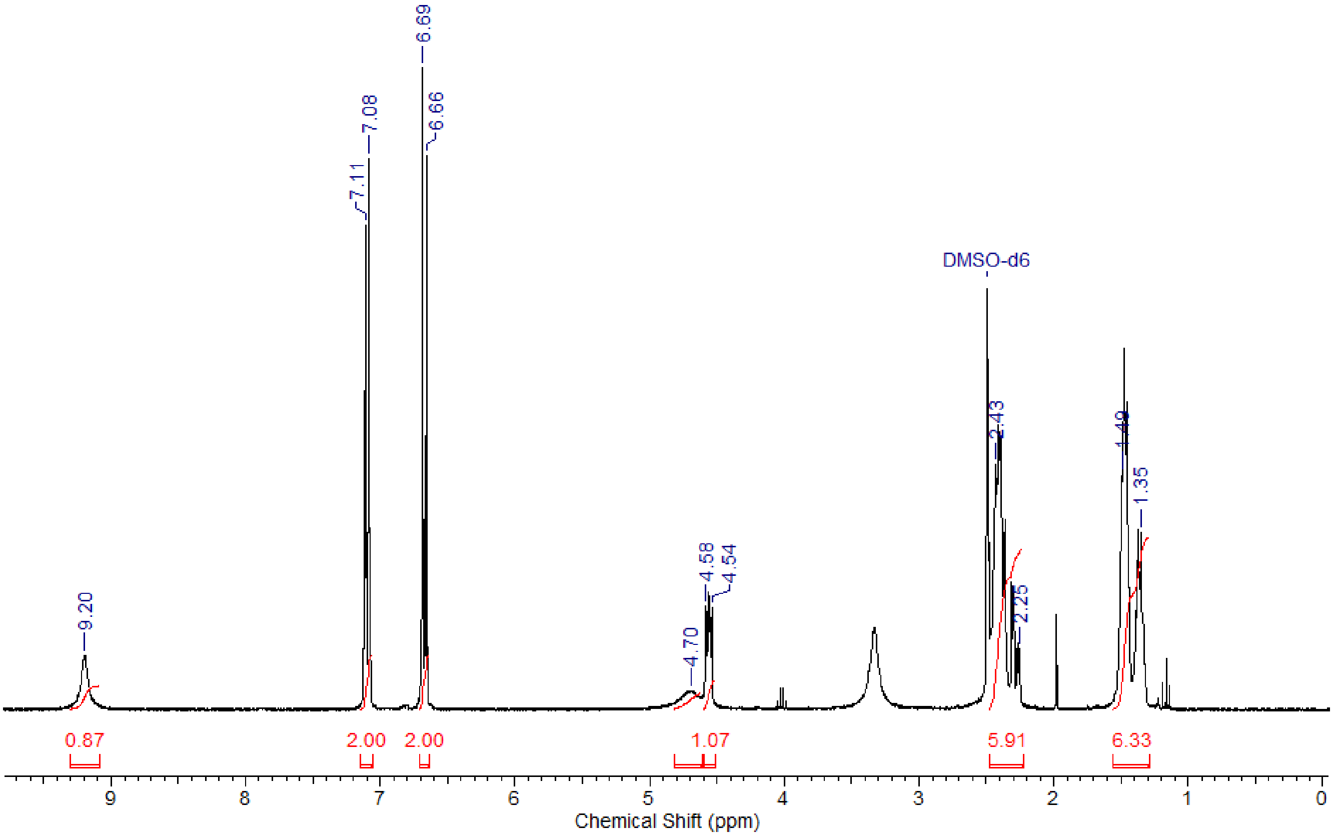

Acquisition Time (sec)	2.8901	Comment	Imported from UXNMR.		
Date	12 Apr 2019 06:01:00			File Name	D:\Институт\Спектры_5224001r
Frequency (MHz)	75.47	Nucleus	13C	Number of Transients	64
Original Points Count	65536	Points Count	131072	Pulse Sequence	zgpg
Solvent	DMSO-D6	Sweep Width (Hz)	22675.74	Temperature (degree C)	25.160

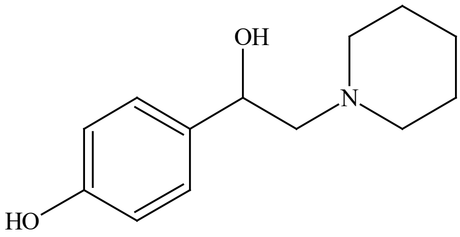

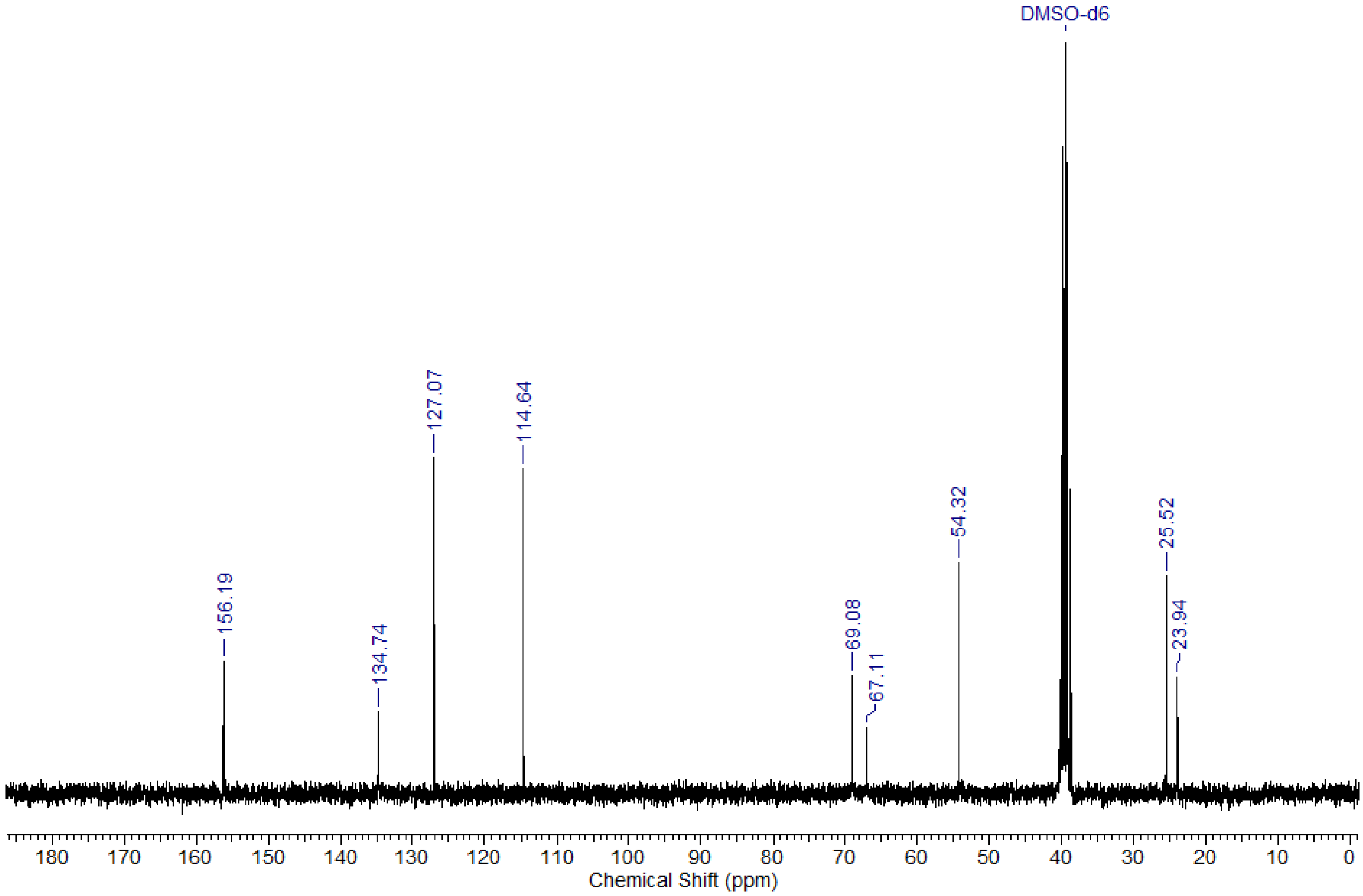

Supplement: Supplementary file 1 [file ijms-25-08904-s001.zip › Zhidkova et al Supplementary Figure 2 Revised.pdf]
