# Supplementary material for: Synthesis and Anti-Cancer Activity of the Novel Selective Glucocorticoid Receptor Agonists of the Phenylethanolamine Series
Source: Int J Mol Sci. 2024 Aug 15;25(16):8904. doi: 10.3390/ijms25168904 (PMC11354514; doi:10.3390/ijms25168904)
Supplement: Supplementary file 1 [file ijms-25-08904-s001.zip › Zhidkova et al Supplementary Figure 6 Revised.pdf]

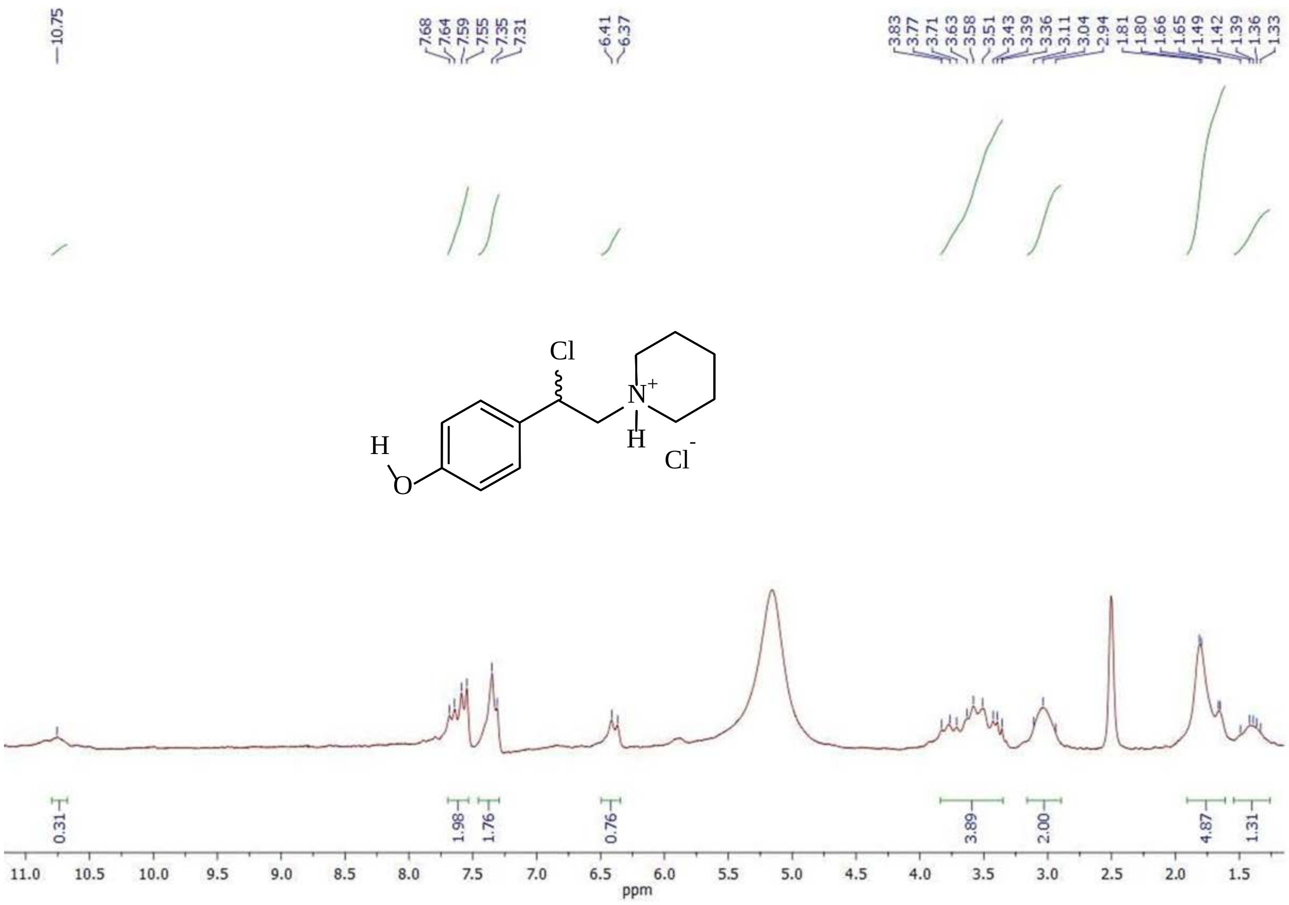

Supplementary Figure 6. Copy of ^{1}H NMR spectra of 1-(2-chloro-2-(4-hydroxyphenyl)ethyl)piperidin-1-ium chloride (CpdA-04). The structures of compounds were established using 1D NMR (1H, 13C) spectroscopy on Bruker 300 spectrometers at 293 (see details in “Materials and Methods”)
