# Supplementary material for: Synthesis and Anti-Cancer Activity of the Novel Selective Glucocorticoid Receptor Agonists of the Phenylethanolamine Series
Source: Int J Mol Sci. 2024 Aug 15;25(16):8904. doi: 10.3390/ijms25168904 (PMC11354514; doi:10.3390/ijms25168904)
Supplement: Supplementary file 1 [file ijms-25-08904-s001.zip › Zhidkova et al Supplementary Table 1 Revised.pdf]

Supplementary Table 1. Structure and characterization of CpdA-01-08.

Compound	Structure	Characterization
1-(2-chloro-2-(4-methoxyphenyl)ethyl)piperidin-1-ium chloride (CpdA-01)	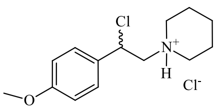	Colorless powder, 99% yield (858 mg). ^{1}H NMR (300 MHz, DMSO-d6): δ = 1.35-1.42 (m, 1H, CH_2); 1.65-1.79 (m, 5H, CH_2); 2.99-3.01 (m, 2H, CH_2); 3.37-3.60 (m, 4H, CH_2); 3.74 (s, 3H, OCH_3); 6.28-6.30 (m, 1H, CH); 6.95-6.98 (m, 2H, H_{arom}); 7.36-7.40 (m, 2H, H_{arom}); 10.61 (s, 1H, NH). MS (EI): m/z (%) = 253 [M]+. HRMS (ESI-TOF) m/z: $[M + H]^{+}$ Calculated for $C_{14}H_{20}ClNO$: 254.1306; found: 254.1317.
1-(4-methoxyphenyl)-2-(piperidin-1-yl)ethanol (CpdA-02)	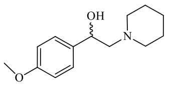	Colorless powder, 71% yield (500 mg). ^{1}H NMR (300 MHz, $CDCl_3$) δ = 1.49 (dd, J = 11.3, 5.6 Hz, 2H, CH_2); 1.61-1.67 (m, 4H, CH2); 2.37-2.45 (m, 4H, CH_2); 2.70-2.76 (m, 2H, CH_2); 3.81 (s, ^{3}H, OCH_3); 4.69 (dd, J = 9.8, 4.3 Hz, 1H, CH); 6.89 (d, J = 8.6 Hz, 2H, H_{arom}); 7.26-7.32 (m, 2H, H_{arom}). Anal. Calculated for $C_{14}H_{21}NO_2$: C, 71.38; H, 9.00; N, 5.85. Found: C, 71.46; H, 8.99; N, 5.95. Lit. [S1].
4-(1-hydroxy-2-(piperidin-1-yl)ethyl)phenol (CpdA-03)	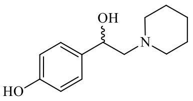	Colorless powder, 33% yield (219 mg). ^{1}H NMR (300 MHz, DMSO-d6): δ = 1.32-1.39 (m, 2H, CH_2); 1.46-1.47 (m, 4H, CH_2); 2.24-2.44 (m, 6H, CH_2); 4.54-4.56 (m, 1H, CH); 6.66-6.69 (m, 2H, H_{arom}); 7.09-7.12 (m, 2H, H_{arom}); 9.18 (s, 1H, OH). Anal. Calculated for $C_{13}H_{19}NO_2$: C, 70.45; H, 8.65; N 6.20, Found: C, 70.56; H, 8.65; N, 6.33. Lit. [S2].
1-(2-chloro-2-(4-hydroxyphenyl)ethyl)piperidin-1-ium chloride (CpdA-04)	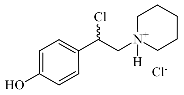	Colorless powder, 99% yield (817 mg). ^{1}H NMR (300 MHz, DMSO-d6): δ = 1.33-1.49 (m, ^{1}H, CH_2); 1.65-1.81 (m, 5H, CH_2); 2.94-3.11 (m, 2H, CH_2); 3.36-3.839 (m, 4H, CH_2); 6.37-6.41 (m, 1H, CH); 7.31-7.35 (m, 2H, H_{arom}); 7.55-7.68 (m, 2H, H_{arom}); 10.75 (s, 1H, NH). MS (EI): m/z (%) = 203 $[M-HCl]^{+}$.
1-(4-methoxyphenyl)-2-(methylamino)ethanol (CpdA-05)	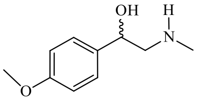	Colorless powder, 33% yield (60 mg). ^{1}H NMR (300 MHz, DMSO-d6): δ = 2.29 (s, ^{3}H, CH_3); 2.50-2.56 (m, 2H, CH_2); 3.73 (s, 3H, OCH3); 4.53-4.59 (m, 1H, CH); 6.87 (d, J = 8.40 Hz, 2H, H_{arom}); 7.24 (d, J = 8.40 Hz, 2H, H_{arom}). MS (EI): m/z (%) = 181 $[M]^{+}$. Anal. Calculated for $C_{10}H_{15}NO_2$: C, 66.13; H, 8.42; N, 7.35. Found: C, 66.27; H, 8.34; N, 7.73. Lit. [S3].
2-chloro-2-(4-methoxyphenyl)-N-methylethanammonium chloride (CpdA-06)	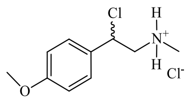	Colorless powder, 33% yield (233 mg). ^{1}H NMR (300 MHz, DMSO-d6): δ = 2.63 (s, 3H, CH_3); 3.76 (s, 5H, OCH_3+CH_2); 6.13-6.19 (m, 1H, CH); 7.00 (d, J = 8.06 Hz, 2H, H_{arom}); 7.41 (d, J = 8.06 Hz, 2H, H_{arom}); 9.29 (s, 2H, NH). MS (EI): m/z (%) = 199 $[M]^{+}$.
1-(3,4-dimethoxyphenyl)-2-(methylamino)ethanol (CpdA-07)	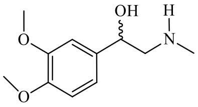	Colorless powder, 63% yield (133 mg). ^{1}H NMR (300 MHz, $CDCl_3$) δ = 2.44 (s, 3H, CH_3); 2.66-2.80 (m, 2H, CH_2); 2.88 (s, 2H, OH+NH); 3.86 (s, 3H, OCH_3); 3.88 (s, 3H, OCH_3);4.67-4.72 (dd, J = 8.4, 4.3 Hz, 1H, CH); 6.84 (s, 1H, H_{arom}); 6.86-6.87 (m, 1H, H_{arom}); 6.93-6.94 (m, 1H, H_{arom}). MS (EI): m/z (%) 211 $[M]^{+}$. HRMS (ESI-TOF) m/z: $[M + H]^{+}$. Calculated for $C_{11}H_{17}NO_3$: 212.1281; found: 212.1275. Lit. [S3].
2-chloro-2-(3,4-dimethoxyphenyl)-N-methylethanammonium chloride (CpdA-08)	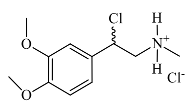	Colorless powder, 33% yield (262 mg). ^{1}H NMR (300 MHz, DMSO-d6) δ = 2.62 (s, ^{3}H, CH_3); 3.74 (s, 4H, $CH^{+}OCH_3$); 3.76 (s, ^{4}H, CH+OCH_3); 6.12-6.16 (m, 1H, CH); 6.98 (s, 2H, H_{arom}); 7.06 (s, 1H, H_{arom}); 9.21 (s, 2H, NH). MS (EI): m/z (%) = 198 $[M - MeO]^{+}$.
