# Supplementary material for: Synthesis and Anti-Cancer Activity of the Novel Selective Glucocorticoid Receptor Agonists of the Phenylethanolamine Series
Source: Int J Mol Sci. 2024 Aug 15;25(16):8904. doi: 10.3390/ijms25168904 (PMC11354514; doi:10.3390/ijms25168904)
Supplement: Supplementary file 1 [file ijms-25-08904-s001.zip › Zhidkova et al Supplementary Table 2 Revised.pdf]

Supplementary Table 2. MolDock scores for Dex, CpdA, CpdA-01-08

Ligand	MolDock score	Rerank score	HBond
CpdA	-85.0357	-69.2085	-9.5861
CpdA-01	-89.6538	-54.7185	-5
CpdA-02	-94.8973	-77.1057	-2.45295
CpdA-03	-87.694	-71.816	-5.70635
CpdA-04	-94.8761	-55.8118	-7.97969
CpdA-05	-80.0126	-57.9591	-2.5
CpdA-06	-83.5671	-67.1426	-1.86454
CpdA-07	-84.9843	-73.3817	-6.92932
CpdA-08	-90.157	-72.1682	-2.5
Dex	-145.589	-128.115	-11.8139
