# Supplementary material for: Synthesis and Anti-Cancer Activity of the Novel Selective Glucocorticoid Receptor Agonists of the Phenylethanolamine Series
Source: Int J Mol Sci. 2024 Aug 15;25(16):8904. doi: 10.3390/ijms25168904 (PMC11354514; doi:10.3390/ijms25168904)
Supplement: Supplementary file 1 [file ijms-25-08904-s001.zip › Zhidkova et al Supplementary Table 3 Revised.pdf]

Supplementary Table 3. Primer sets for Q-PCR analysis

**Gene symbol**	**Sequence**	
	**Forward 5’-3'**	**Reverse 5’-3'**
Rpl27	ACCGCTACCCCCGCAAAGTG	CCCGTCGGGCCTTGCGTTTA
FKBP51	GAATGGTGAGGAAACGCCGAT	TGCCAAGACTAAAGACAAATGGT
GILZ	AACAACGAAATGTATCAGACCC	TGTCCAGCTTAACGGAAACCA
IL1	ATGATGGCTTATTACAGTGGCAA	GTCGGAGATTCGTAGCTGGA
IL6	GGGAGCGATAAACACAAACTCTGC	GAGAAGGCAACTGGACCGAAG
CCND1	AGACCTTTGTGGCCCTCTGTG	CAGTCCGGGTCACACTTGAT
CCND2	CTCAGACCTTCATTGCTCTGTGT	CTCAGTCAGGGCATCACAAGT
